# A Carbon-14 Beta-Ray Standard, Benzoic Acid-7-C^14^ in Toluene, for Liquid Scintillation Counters

**DOI:** 10.6028/jres.064A.013

**Published:** 1960-04-01

**Authors:** W. F. Marlow, R. W. Medlock

## Abstract

A carbon-14 beta-ray standard for use in liquid scintillation counting has been prepared and standardized. The sample consists of benzoic acid-7-C^14^ dissolved in toluene. Samples of the solution were oxidized quantitatively in a Paar oxygen bomb, and the radioactivity of the carbon dioxide was compared with the radioactivity of carbon dioxide prepared quantitatively from the Bureau’s sodium carbonate-C^14^ standard.

## 1. Introduction

With the greatly increased use of liquid-scintillation counting as a means of measuring carbon-14 activity, particularly in biochemical and medical studies, the need has also arisen for a suitable carbon-14 standard sample. Since the solvent in most liquid-scintillation systems is generally toluene or xylene, the standard sample should be readily soluble in, and compatible with toluene or xylene. Benzoic acid-7-C^14^ dissolved in toluene was decided upon as a suitable sample.

It was decided to standardize the sample by quantitative oxidation of the benzoic acid and toluene, with quantitative collection of the carbon dioxide produced, followed by measurement of the level of radioactivity of this carbon dioxide in an ionization chamber.

It has been reported by W. D. Armstrong and coworkers [1]^1^ that C^14^O_2_ and C^12^O_2_ are evolved at different rates during oxidation of organic compounds. They report that on wet combustion of xanthydrourea (using the Van Slyke-Folch oxidizing mixture), the C^14^O_2_ came off to a very small extent in the first portions of reactant gasses and to a very large extent in the latter portions. Severe treatment was necessary to effect complete combustion; a small amount of nonoxidized material could, therefore, result in a disproportionately large loss of C^14^O_2_. When the same compound was oxidized in a stream of oxygen (Pregl’s method), it was found that more C^14^O_2_ came off in the first half of the reaction than in the last half.

In order to avoid the possibility of error due to this possible isotope effect in either method, it was decided to use a method certain to effect complete combustion of the sample and complete mixing of the C^12^O_2_ and C^14^O_2_ produced. After consultation with personnel of the Bureau’s Thermochemistry Section, it was decided to burn the sample in a modified Paar oxygen bomb and separate the carbon dioxide produced from the oxygen and water.

## 2. Preparation of the Standard Sample

The benzoic acid-7-C^14^ used was prepared by the Bio-Organic Group, Radiation Laboratory, University of California at Berkeley, Calif. The procedure consisted of the carbonation of phenyl magnesium bromide, ether extraction from the reaction mixture, extraction from the ether layer with sodium carbonate solution, and concentration, neutralization, and crystallization.

**Figure 1:**
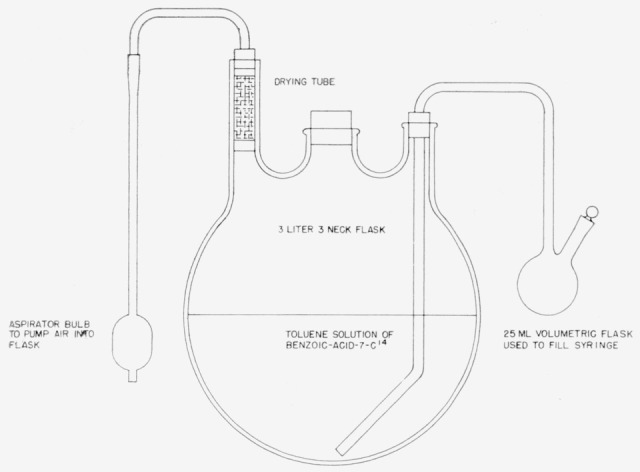
Reservoir for toluene solution of benzoic acid-7–*C*^14^.

**Figure 2:**
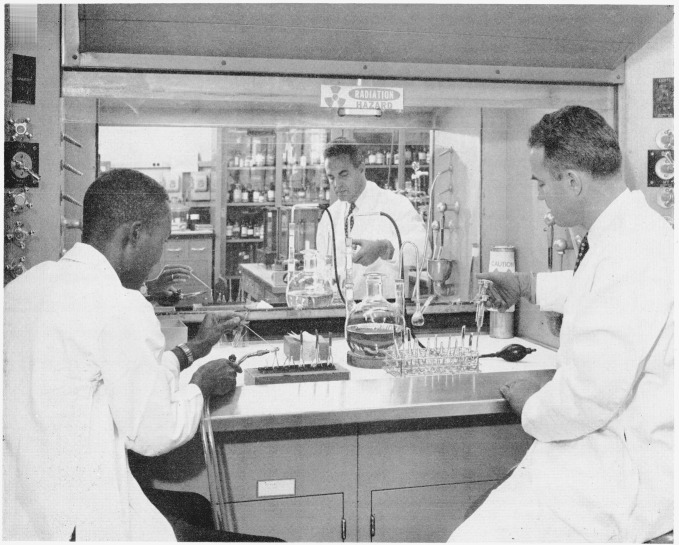
Setup and procedure for filling and sealing ampoules with benzoic acid-7–*C*^14^.

Seventy milligrams of the dry benzoic acid-7-C^14^, containing approximately 0.77 mc of C^14^ were placed in a dry 3-neck 3,000-ml round bottom flask protected from the atmosphere by a magnesium perchlorate—Ascarite trap. Reagent-grade toluene that had been dried over sodium was then distilled from freshly added sodium, and 1,500-ml of the fraction boiling between 109.8° and 110.6° C were distilled directly into the 3,000-ml round bottom flask. The mixture was thoroughly agitated to effect a homogeneous solution. The flask was then set up as shown in figure 1. A 5-ml syringe with hypodermie needle, set to deliver 3 ml, was used to transfer the toluene solution of benzoic acid-7-C^14^ to 500 round bottom glass ampoules. The ampoules were cooled in a dry ice—alcohol—water bath (−30° C) and flame-sealed. Figure 2 shows apparatus and techniques used.

## 3. Combustion of the Sample and Collection of the Carbon Dioxide

Samples of the contents of several of the individual ampoules were taken for analysis after the ampoules had been sealed at least 6 months. This elapsed time presumably allowed any adsorption of benzoic acid on the walls of the ampoules to reach equilibrium. This was verified by the agreement obtained on analyses of contents of ampoules performed 6, 10, and 12 months after preparation.

F. N. Hayes [2, 3] reported that no apparent adsorption effects occurred in liquid-scintillating counting with benzoic acid-7-C^14^ if the total concentration of benzoic acid was above 2 mg/liter; below this value, adsorption apparen tly became serious. Hayes also reported that concentrations of benzoic acid above approximately 1,300 mg/liter apparently had a quenching effect in liquid-scintillation counting. With the amount of carrier benzoic acid used in the present samples (total concentration of benzoic acid was approximately 47 mg/liter), neither adsorption nor quenching effects will be present according to Hayes’ data.

**Figure 3:**
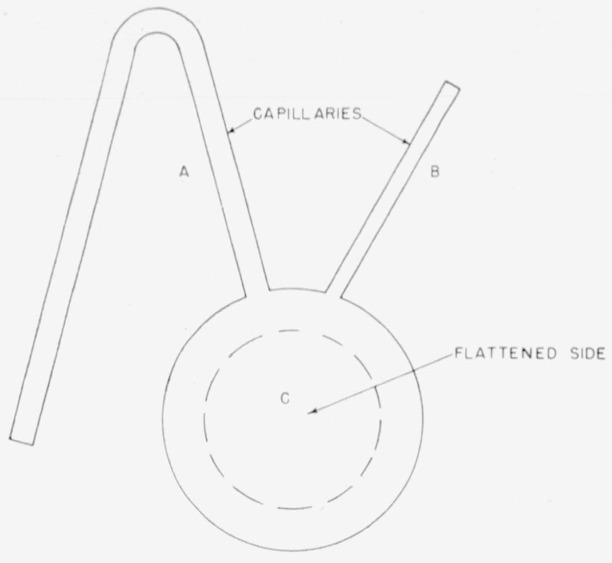
Glass ampoule for sampling toluene solution of benzoic acid-7–*C*^14^ and introducing it into Paar bomb.

The standard solution was diluted quantitatively with 1 or 2 parts, by weight, of pure toluene before sampling; this was done to obtain a solution of low enough specific activity so that a large enough sample could be taken to give sufficient carbon dioxide for analysis without yielding too much radioactivity to be measured in the ionization chamber. The ampoule used to weigh and introduce the samples into the Paar bomb is shown in figure 3. The sample was drawn in through capillary *A* by applying very mild suction to capillary *B*; capillary *A* and then *B* were sealed, using a pinpoint oxygen-gas flame. The ampoule had to be completely filled, since any air bubble in the sealed ampoule could cause the ampoule to break in the Paar bomb as the oxygen pressure was introduced. The ampoules used held from 0.12 to 0.21 g of the sample.

**Figure 4:**
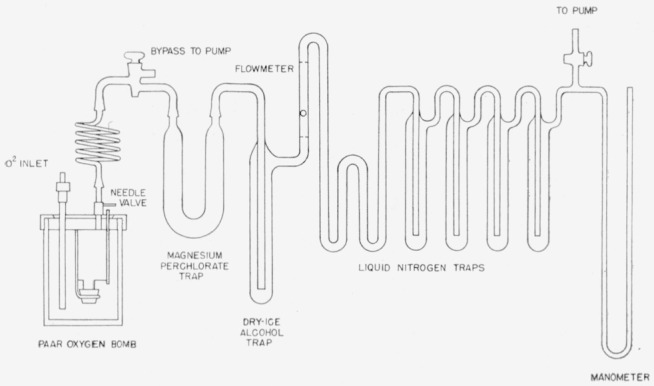
System for collection of *CO*_2_ from combustion products in Paar bomb.

**Figure 5:**
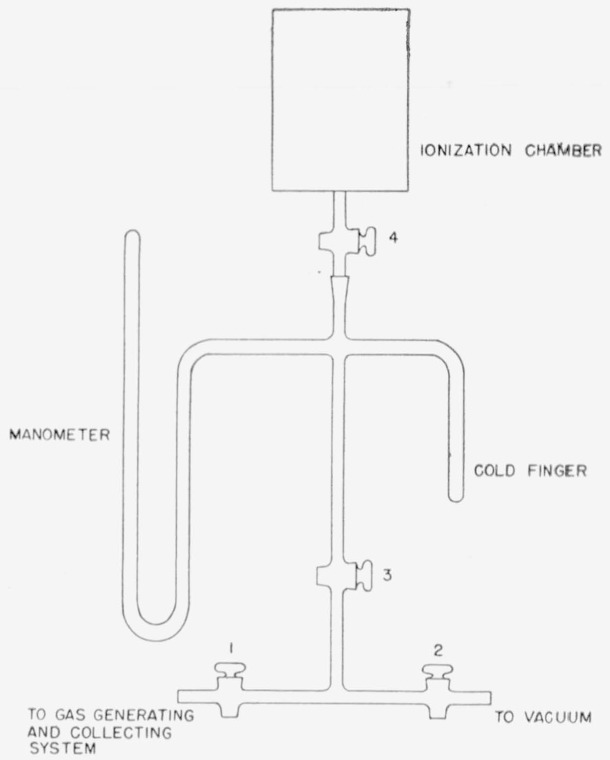
Gas measuring system.

After the combustion, the gases were bled very slowly through a magnesium perchlorate trap, dry ice—alcohol trap, flowmeter, two liquid nitrogen traps, where the carbon dioxide was frozen out, and finally out through a vacuum pump (see fig. 4). The exit pressure was kept between 70 and 80 mm Hg for best results. When all the gases had been removed from the bomb and passed through the traps, as evidenced by zero flow rate on the flow meter and minimum pressure on the barometer, all of the carbon dioxide was frozen in to one of the liquid nitrogen traps and the trap pumped down for 15 to 20 min to less than 1 mm Hg pressure. The carbon dioxide was then transferred to the calibrated gas-collecting and measuring system, figure 5, where its volume and pressure were measured to determine the yield. Nonradioactive carbon dioxide was added to bring the gas to the desired pressure (1 atm) and the gases thoroughly mixed by repeated freezing and expanding of the carbon dioxide. A known fraction was collected in a 250-ml ionization chamber.

## 4. Measurement of Radioactivity of the CO_2_

The ionization chamber containing the known portion of the collected carbon dioxide was placed on the electrometer head of a vibrating reed electrometer and the drift rate measured. After correcting for background, the net drift rate was compared with the net drift rate of carbon dioxide, at the same pressure, quantitatively generated from a National Bureau of Standards sodium carbonate-C^14^ beta-ray standard, and the activity was calculated.

## 5. Calculations and Results

**Figure 6:**
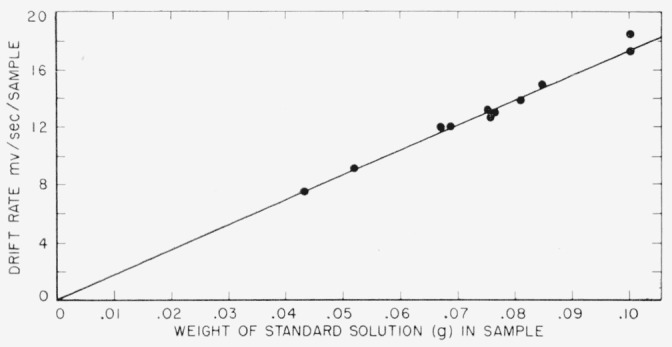
Graph showing drift rate on vibrating reed electrometer for *CO*_2_ from toluene solution of benzoic acid-7–*C*^14^ versus weight of sample of toluene solution of benzoic acid-7–*C*^14^.

The data and calculations are given in table 1 and in the graph, figure 6.

The graph, with the drift rate in millivolts per second per sample plotted versus the weight of the sample shows the reproducibility of the method over the range of sample weights used.

Standard deviation:

$$\begin{array}{l} \sigma =\sqrt{\frac{\sum d^{2}}{n\left(n-1\right)}}=\sqrt{\frac{211.43}{110}}=\sqrt{1.92}=\pm 1.386\\ \;1.386/173.72\;=\;\pm \;0.80\;\text{percent}. \end{array}$$

Therefore, the drift rate for the carbon dioxide obtained from the combustion of the benzoic acid-7-C^14^ in toluene, under the conditions used here, was expressed as 173.7 ± 0.8 percent mv/sec/g standard.

The drift rate for the carbon dioxide obtained the quantitative conversion of the sodium carbonate-C^14^ standard had been determined to be 13.18 mv/sec/g standard when measured under the same conditions as for the carbon dioxide from the benzoic acid. The activity of the sodium carbonate-C^14^ standard has been determined to be 1250 ± 1.5 percent dps/g by absolute counting of the carbon dioxide obtained from the quantitative conversion of the sodium carbonate.

Therefore, the activity of the benzoic acid-7-C^14^ in toluene is calculated:

$$\frac{\mathrm{mv}/\text{sec}/g\;\text{benzoic}\;\text{acid}\;\text{standard}}{\mathrm{dps}/g\;\text{benzoic}\;\text{acid}\;\text{standard}}=\frac{{\mathrm{mv}/\text{sec}/g\;\mathrm{Na}}_{2}{\mathrm{CO}}_{3}\;\text{standard}}{{\mathrm{dps}/g\;\mathrm{Na}}_{2}{\mathrm{CO}}_{3}\;\text{standard}}$$

$$\mathrm{dps}/g\;\text{benzoic}\;\text{acid}\;\text{standard}=\frac{173.7\times 1250}{13.18}=16.47\times 10^{4}.$$

The value assigned was 16.5 × 10^4^ dps/g of solution, with a precision of ± 1 percent on comparison with sodium carbonate-C^14^ standard, whose value is known with an accuracy of ± 1.5 percent.

## Figures and Tables

**Table 1:** 

Run	Wt of sample	*X*	*Y*	*Y/X*	$\left(\bar{Y/X}\right)-\left(Y/X\right)$
		Wt of standard in sample	mv/sec/sample	mv/sec/g standard	Deviations(*d*)
					
	*g*	*g*			
1	0.1545	0.0519	9.213	177.44	+3.72
2	.2047	.0687	12.076	175.68	+1.96
3	.1996	.0670	11.864	177.00	+3.28
4	.2044	.1011	18.345	181.45	+7.73
5	.1520	.0752	13.199	175.62	+1.90
6	.2012	.1012	17.025	168.25	−5.49
7	.1682	.0846	14.829	175.20	+1.48
8	.1508	.0759	12.693	167.28	−6.44
9	.1513	.0761	12.788	167.98	−5.74
10	.1611	.0811	13.803	170.28	−3.44
11	.1287	.0433	7.557	174.74	+1.02

$\left(\bar{Y/X}\right)=173.72$					

## Footnotes

1 Figures in brackets indicate the literature references at the end of this paper.
